# Developing and Assessing the Performance of a Machine Learning Model for Analyzing Drinking Behaviors in Minipigs for Experimental Research

**DOI:** 10.3390/s26020402

**Published:** 2026-01-08

**Authors:** Frederik Deutch, Lars Schmidt Hansen, Firas Omar Saleh, Marc Gjern Weiss, Constanca Figueiredo, Cyril Moers, Anna Krarup Keller, Stefan Rahr Wagner

**Affiliations:** 1 Department of Clinical Medicine, Aarhus University, 8000 Aarhus, Denmark; 2 Department of Urology, Aarhus University Hospital, 8000 Aarhus, Denmark; 3 Department of Electro- and Computer Technology, Section of Biomedical Engineering, Aarhus University, 8000 Aarhus, Denmark; 4 Institute of Transfusion Medicine and Transplant Engineering, Hannover Medical School, 30625 Hannover, Germany; 5 Department of Surgery, University Medical Center Groningen, University of Groningen, 9713 GZ Groningen, The Netherlands

**Keywords:** machine learning, monitoring, drinking behavior, minipigs, experimental animals, animal welfare, sensor-based surveillance

## Abstract

Monitoring experimental animals is essential for ethical, scientific, and financial reasons. Conventional observation methods are limited by subjectivity and time constraints. Camera-based monitoring combined with machine learning offers a promising solution for automating the monitoring process. This study aimed to validate and assess the performance of a machine learning model for analyzing drinking behavior in minipigs. A novel, vision-based monitoring system was developed and tested to detect drinking behavior in minipigs. The system, based on low-cost Raspberry Pi units, enabled on-site video analysis. A dataset of 5297 images was used to train a YOLOv11n object detection model to identify key features such as pig heads and water faucets. Drinking events were defined by the spatial proximity of these features within video frames. The multi-class object detection model achieved an accuracy of above 97%. Manual validation using human-annotated ground truth on 72 h of video yielded an overall accuracy of 99.7%, with a precision of 99.7%, recall of 99.2%, and F1-score of 99.5%. Drinking patterns for three pigs were analyzed using 216 h of video. The results revealed a bimodal drinking pattern and substantial inter-pig variability. A limitation to the study was chosen methods missing distinguishment between multiple pigs and the absence of quantification of water intake. This study demonstrates the feasibility of a low-cost, computer vision-based system for monitoring drinking behavior in individually housed experimental pigs, supporting earlier detection of illness.

## 1. Introduction

Animal welfare in research should be considered to ensure reliable scientific results and for ethical and financial reasons [1,2]. When measuring animal welfare in experimental settings, in-person assessments may be affected by human subjectivity and the presence of humans in itself [3]. Furthermore, these assessments rely on the physical presence of caretakers, which restricts observations to a limited part of the day. Automated sensor or vision-based systems can reduce these limitations by replacing the human observer with continuous monitoring that can alert caretakers in case of behavioral irregularities, which may indicate disease [4]. Several activities are relevant to track in experimental pigs, including movement, sedentary behavior, drinking behavior, eating behavior, and play behavior [5].

Machine learning and computer vision have been applied to several species in experimental and agricultural contexts. In mice, vision-based behavioral analysis has supported models estimating frailty indices [6]. In cattle, drinking behavior has been detected from videos using pose estimation combined with temporal classification models [7]. In pigs, recent studies have applied YOLO-based detectors, attention mechanisms, and lightweight architectures for behavior recognition in farm environments [8,9,10,11]. These studies include the detection of lying, standing, eating, drinking, and aggressive interactions. Automated tracking systems for group-housed pigs have also been developed using real-time multi-object tracking [12]. These studies indicate that vision-based monitoring can identify welfare-related behavior in livestock.

Pigs, particularly Göttingen minipigs, offer advantages in translational biomedical research due to physiological similarity to humans and their stable size across developmental stages [13,14,15]. Drinking behavior in healthy pigs follows a consistent diurnal pattern, and deviations from this pattern can indicate illness [4]. This is relevant in survival studies where continuous post-operative monitoring is required, for example, in organ transplantation research.

Wang et al. (2022) [16] provide a review of research progress in vision-based artificial intelligence in smart pig farming, an area related to experimental animal research facility monitoring, including camera-based approaches. Specifically, they investigate the state of the art on the “recognition of pig drinking behavior”. They emphasize that the most common way is the use of RFID tags, with one tag placed on the pig’s ear and receivers installed next to the drinking fountains. This method is costly in terms of infrastructure and invasive due to the need to punch electronic tags into the ears of the pigs. Wang et al. find that a camera combined with machine vision technology can support automated recognition of pig drinking behavior. However, none of the reported studies were used for real-time tracking of animals.

Reza et al. (2025) [17] provide a recent review on RGB imaging technologies using cameras and sensors for enhanced pig disease symptoms and find that the predominant image sensor devices in use for animal monitoring are standard digital and surveillance cameras, which capture visible light for generating color and/or grayscale images. This includes various types of charge-coupled devices and cameras, such as infrared, depth, and three-dimensional (3D) cameras. Also, they find that 3D, multispectral, and hyperspectral image cameras tend to be costly. The review primarily focuses on RGB cameras, which are extensively utilized in various studies conducted on pig farms but does not report on usage in experimental animal facilities. Like Wang et al., they also report on several types of use cases, incl. drinking behavior.

Focusing on camera-based detection of drinking behavior, we find a series of existing recent studies. This includes Kashiha et al. in 2013 [18] who used the distance between the key points of a pig outline image and the center of mass to judge the occurrence of pig drinking behavior utilizing video recordings; this was not evaluated in a real-time use case.

Yang et al. (2018) [19] used the “image occupation index” to improve the recognition performance of pig drinking behavior recognition using YOLOv2, achieving 96.49% drinking behavior recognition, but did not report on whether this was achieved in real-time (Chinese version only).

Chen et al. (2020) [20] extracted spatial features based on the ResNet-50 model and used long short-term memory (LSTM) to identify pig drinking behavior. The results show that this method can detect pig drinking behavior. However, the study did not focus on real-time usage but instead used video recordings.

Alameer et al. (2020) [21] used Microsoft Kinect 3D cameras (Microsoft Kinect for Xbox One, Microsoft, Redmond, DC, USA) mounted to the ceiling to automate the recognition of postures and drinking behavior in pigs. The cameras covered a large space of the pen, including the drinking sources. Videos of pig behavior were recorded at 25 frames per second with an image frame of 640 pixels × 360 pixels. They achieved a precision of 0.98 for drinking behavior. However, the price and suitability of 3D cameras is a concern, and the possibility of using the system for real-time detection of drinking behavior was not discussed.

Zhuang et al. (2023) [22] achieved high recall on tracking drinking behavior using cameras only (94–100%), but this was within the confines of a combined feeding and drinking station, and would thus require additional infrastructure for each drinking nipple.

In conclusion, several studies investigate the use of cameras and vision-based tracking for monitoring the drinking behavior of pigs in pig farms. However, no studies focus on experimental minipigs located in research facilities, using low-cost equipment for local and real-time processing of the video for monitoring and alerting.

A single study by Tagoe et al. (2024) [23] utilizes a Raspberry Pi unit with a camera for the real-time tracking of pigs, but does not investigate drinking behavior. The project involves a centroid-based tracking algorithm that measures movement by calculating Euclidean distances between centroids across frames. Data gathered on individual pig movements in meters is analyzed and visualized through time series graphs, providing valuable insights into the motion of individual pigs.

Although previous work has addressed automated drinking behavior monitoring in commercial group-housed farm pigs or other livestock species [16,17,18,19,20,21,22,23,24,25,26,27], no published studies have examined camera-only monitoring of drinking behavior in minipigs living in single-pig pens using low-cost Raspberry Pi units with on-device machine learning in a research facility setting.

This study builds on the PigSpies system, which previously enabled posture classification and activity-level tracking of pigs in an experimental setting [13]. Here, we extend this system by integrating automated drinking behavior detection to support identification of irregular drinking patterns in a cost-effective and sustainable way, which closes the gap regarding low-cost real-time monitoring of minipigs in an experimental facility.

The aim of this study is to validate a vision-based machine learning model for real-time classification of drinking behavior in Göttingen minipigs and to provide descriptive insight into drinking patterns in healthy individuals using low-cost Raspberry Pi 4 computers equipped with low-cost commercial cameras.

## 2. Materials and Methods

### 2.1. Experimental Site

The research was conducted at an animal facility at Aarhus University, Denmark, where Göttingen minipigs were housed, with trained personnel handling the daily care of the pigs. The pigs were a crossbreed of three different pig breeds and are widely used in medical research due to their human-like psychology and their slow growth. Fully grown minipigs weigh around 40 kg. The slow growth of the pigs ensures that the video analysis was not disturbed by changes in pig size or the necessity to expand the pen size.

The study was performed on healthy pigs before any medical or pharmaceutical interference to ensure reliable results. The subjects were kept in neighboring, but single-occupancy, pens with dimensions of 240 cm × 266 cm. Consistent environmental conditions were maintained for the duration of the study, which was achieved through the implementation of an automated 12 h light–dark cycle (lights on from 06:30 to 18:30) with a fixed luminance level, automatic temperature and humidity control, and daily management by trained personnel.

### 2.2. Component Deployment and Data Collection

A wide-angle 110-degree USB camera (Marhynchus, Shanghai, China) [28] was secured with a metal camera clamp (SMALLRIG Ballhead Clamp, Hong Kong, China) [29] and ceiling-mounted to provide a fixed, top-down view of the enclosure. A Raspberry Pi 4 Model B (4 GB RAM) (Raspberry Pi Foundation, Cambridge, UK) [30] served as the dedicated edge computing node, processing video data locally from a 128 GB SanDisk Extreme SD card (SanDisk, Milpitas, CA, USA).

The recording process was managed by a custom Python version 3.12 service running on each Raspberry Pi. This service automated data capture by employing a context-aware trigger; it initiated video recording only when the facility’s lights were active, corresponding to the 12 h photoperiod. This setup ensured that raw video footage was continuously recorded during periods of animal activity and stored locally for subsequent offline processing and analysis.

### 2.3. Image Pre-Processing

No image pre-processing steps, such as filtering or normalization, were applied in this study. The consistency of the data collection, guaranteed by a static camera and controlled automated lighting, meant that the raw video frames were already optimized for analysis. This removed the need to correct for common visual variations and enabled the object detection model to operate directly on the unprocessed images.

### 2.4. Training Configurations

The annotated dataset of 5297 images was randomly partitioned into three subsets: 70% for training (*n* = 3708), 20% for validation (*n* = 1059), and 10% for testing (*n* = 530). The YOLOv11n model was trained for 100 epochs using a batch size of 16 and an input image resolution of 640 × 640 pixels. Optimization was performed using the default Ultralytics ‘auto’ setting (SGD) with an initial learning rate (lr0) of 0.01, a final learning rate factor (lrf) of 0.01, momentum of 0.937, and weight decay of 0.0005. To enhance model generalization, several data augmentation techniques were applied during training, including Mosaic augmentation (1.0), horizontal flip (0.5), random erasing (0.4), and random adjustments to hue (0.015), saturation (0.7), and value (0.4).

Dataset Annotation: A custom dataset of 5297 images of four different pigs was annotated using the Roboflow platform with six object classes: “Pig-Standing”, “Pig-Laying”, “Pig-Head”, “Water-Faucet”, “Keeper”, and “Feces”. The “Pig-Head” and “Water-Faucet” classes were specifically used by the model for proximity-based drinking detection.

Model Training: A YOLOv11n (nano) model was selected for its efficiency on edge devices. The model was trained for 100 epochs on the custom dataset using the Ultralytics framework within a Google Colab environment.

### 2.5. Proximity Calculation and Drinking Event Detection

A drinking event is not a direct classification from the model but is inferred using a real-time, multi-step algorithm that processes each video frame. The algorithm is designed to identify when a pig’s head is in close and stable proximity to a water source.

Faucet Localization: Upon initialization, the system identifies and fixes the positions of up to two “Water-Faucet” objects with the highest confidence scores. This is done by analyzing the first 30 frames of the video stream. Using fixed faucet locations provides a stable reference point for distance calculations throughout the monitoring session.

Pig Head Detection: In each subsequent frame, the trained YOLOv11n model identifies the bounding boxes and confidence scores for all visible “Pig-Head” objects.

Centroid Calculation: The geometric center (centroid) of each detected “Pig-Head” bounding box and each fixed “Water-Faucet” bounding box is calculated using the following formula:

$$\left(x_{c},y_{c})=(\frac{x_{1}+x_{2}}{2},\;\frac{y_{1}+y_{2}}{2}\right)$$

where $\left(x_{1},y_{1}\right)$ and $\left(x_{2},y_{2}\right)$ are the coordinates of the top-left and bottom-right corners of the bounding box, respectively.

Proximity Normalization: To create a consistent proximity score, the raw pixel distance is normalized relative to the diagonal length of the video frame. The proximity score *P* is calculated as follows:

$$P=1-\frac{d}{d_{diag}}$$

where $d_{diag}$ is the diagonal length of the frame in pixels. A score of 1 indicates perfect overlap of centroids, while scores approaching 0 indicate greater separation.

Event Triggering: A frame is flagged as part of a “drinking event” if the proximity score P for any pig head–faucet pair exceeds a predefined threshold (empirically set to 0.98). A continuous sequence of such flagged frames constitutes a single drinking visit, from which the event duration is calculated.

**Figure 1:**
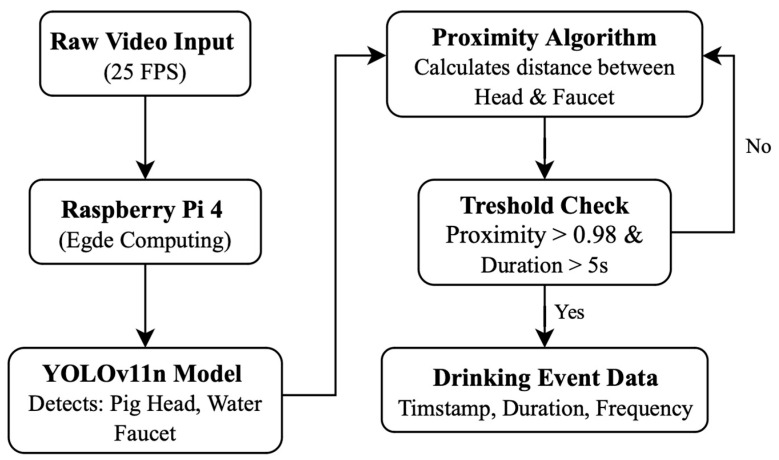
Raw video is continuously captured at 25 FPS and processed locally on a Raspberry Pi 4 for edge computing. A lightweight YOLOv11n object detection model identifies the pig head and water faucet in each frame. A proximity algorithm then calculates the spatial distance between these detected objects. Drinking behavior is inferred using a threshold-based check, requiring both high spatial proximity and a minimum duration. When these conditions are met, a drinking event is registered and stored as structured data, including timestamp, duration, and event frequency.

A methodological diagram illustrating the full processing pipeline is presented in Figure 1.

### 2.6. System Validation

The system’s accuracy was validated by comparing its automated detections against a human-annotated ground truth. A total of 72 h of video footage was manually reviewed. To ensure precise synchronization between the human validator and the model’s output, the original 25 FPS video footage was downsampled during the validation analysis to an effective rate of ~1.38 FPS. This approach yielded a total dataset of 357,388 frames, representing three days (12 h per day) of activity for two different pigs, as seen in Table 1.

**Figure 2:**
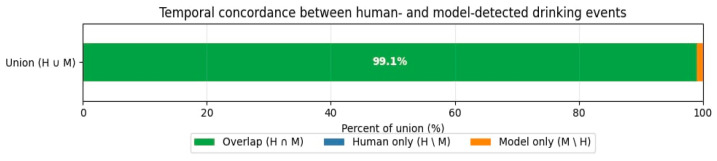
Temporal concordance between human- and model-detected drinking events. An exceptionally high degree of temporal overlap was found. 99.1% of the total time identified as drinking was shared between the human ground truth and the model predictions (Intersection over Union).

Temporal concordance analysis was performed to quantify the extent to which the model’s detected “drinking time” overlapped with the human-annotated “drinking time” based on event registration. Results can be seen in Figure 2.

### 2.7. Characterization of Drinking Behavior

After validation, the model was used to characterize the drinking frequency, duration, and pattern of Göttingen minipigs in stable conditions. A total of 216 h of video material was analyzed to gain better knowledge of the mentioned drinking parameters. The video material consisted of six days of video from three different pigs to ensure a necessary amount of video material and to analyze inter-pig variation. Two of the three were also included in the validation process.

The drinking frequency and pattern were analyzed through drinking event count per hour of the day, while the drinking duration was evaluated by registering the duration in seconds of each drinking event as well as the total drinking duration for each hour of the day. This differentiation was performed to clarify whether drinking events represented substantial water intake or curiosity- and habit-driven visits to the drinking faucets.

## 3. Results

### 3.1. Model Performance

**Figure 3:**
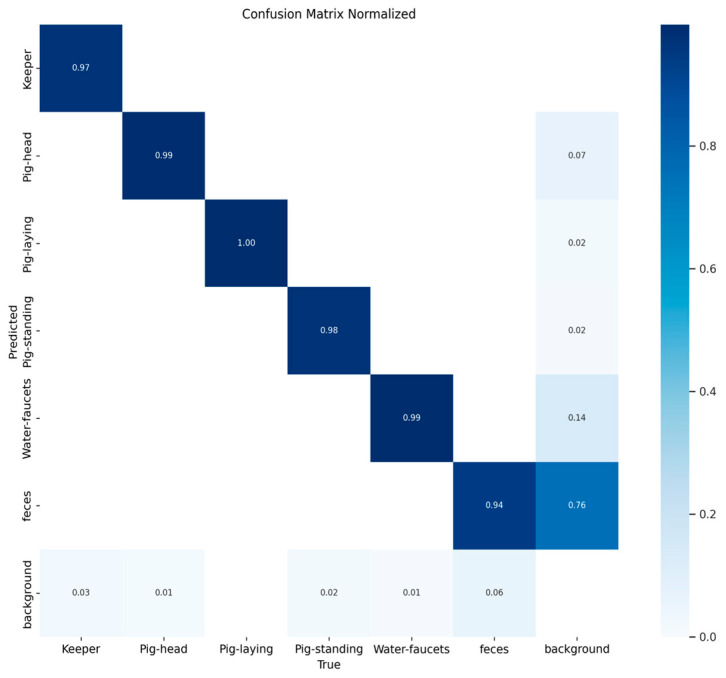
Normalized confusion matrix illustrating the proportional performance of the object detection model on the validation dataset. The values on the diagonal represent the recall for each class, demonstrating high accuracy (≥97%) for all primary objects. The off-diagonal values highlight the main sources of error, with the ‘background’ class being the most frequently misclassified, notably being confused with ‘feces’ (14%) and ‘Pig-head’ (7%). Values of 0.00 are not presented for clarity.

**Figure 4:**
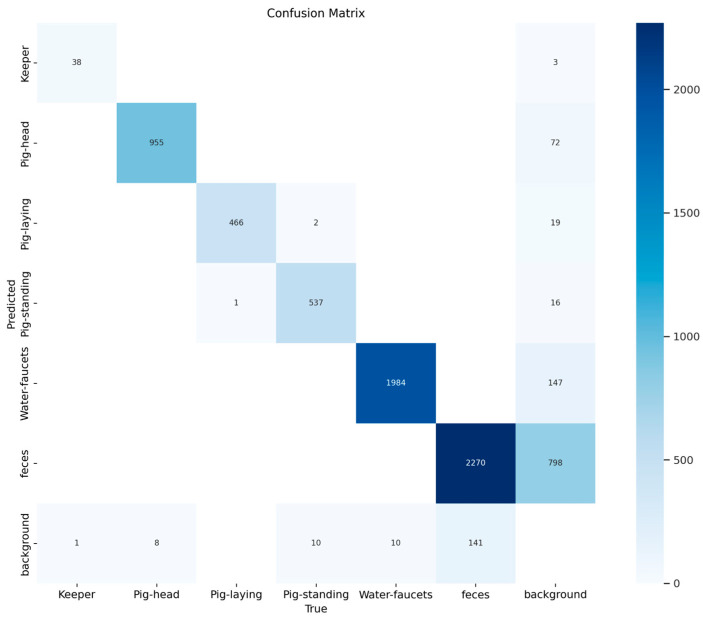
Confusion matrix showing the absolute counts of model predictions versus true labels from the validation set. This matrix quantifies the model’s performance, for example, by correctly identifying 955 ‘Pig-head’ instances and 1984 ‘Water-faucets’ instances. The raw counts provide the exact number of misclassification events, such as the 147 instances where the ‘background’ was incorrectly labeled as ‘feces’.

The performance of the multi-class object detection model on the validation dataset is presented in Figure 3 and Figure 4. The figure includes a normalized matrix showing the proportion of correct predictions (top) and a matrix with the raw prediction counts (bottom).

**Figure 5:**
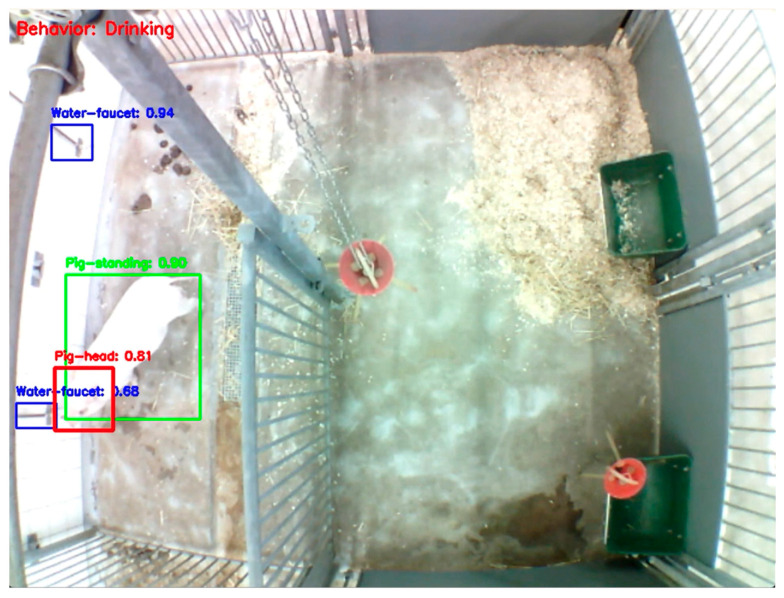
An example of the object detection process. The proximity of the pig head (red) can be seen overlapping with a water faucet (blue) while the model registers “Drinking” as the behavior in the current frame (red text in top left corner). The green box represent the bounding box of the whole pig with characterization of the posture as described in an earlier study [1]. The green box does not have influence of the overlapping of the blue and red boxes, and it does not have any influence on the data output of this study.

The matrices reveal high accuracy for the primary object classes, as indicated by the strong diagonal. The model correctly classified ‘Pig-laying’ in 100% of instances, ‘Pig-head’ and ‘Water-faucets’ with 99% accuracy, and ‘Pig-standing’ and ‘Keeper’ with 98% and 97% accuracy, respectively. The main area of misclassification involved the ‘background’ class, which was correctly identified in 76% of cases but was at times misclassified as ‘feces’ (14%) and ‘Pig-head’ (7%). An illustration of the object detection process can be seen in Figure 5.

### 3.2. Training Metrics

**Figure 6:**
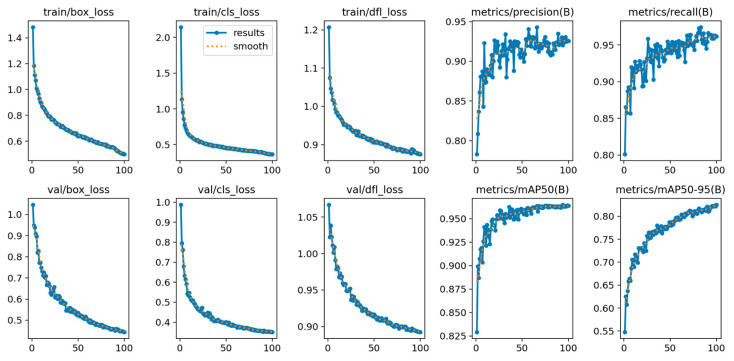
Training and validation learning curves for the object detection model over 100 epochs. The top row displays the metrics for the training set, including decreases in box, classification (cls), and distribution focal loss (dfl), alongside increases in precision and recall. The bottom row shows the corresponding metrics for the validation set, which assess the model’s generalization. Also shown are the mean average precision (mAP) at an IoU threshold of 0.50 and the average mAP across IoU thresholds from 0.50 to 0.95. The consistent decrease in validation loss and the steady increase in mAP indicate successful model convergence. The blue line represents the raw metric values recorded at each epoch, while the dotted orange line represents the smoothed trend (moving average). While the legend is only explicitly displayed in the train/cls_loss subplot to maintain visual clarity and avoid clutter, the underlying data processing is identical for all ten graphs.

Figure 6 depicts the learning curves for the object detection model throughout its 100-epoch training cycle. The metrics are described in Table 2.

The model’s convergence is evident from the steady decrease across all three loss metrics (box, cls, and dfl), reflecting an improved capacity for accurate object localization and classification. Simultaneously, the improvements in precision, recall, and mAP metrics signify a more robust model with a lower rate of false positives and negatives, thereby validating its effectiveness in the task.

### 3.3. Validation Results

**Figure 7:**
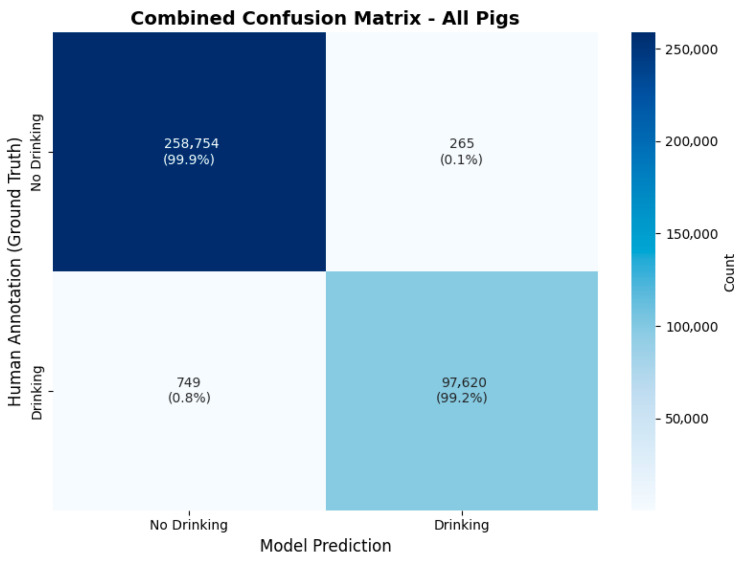
Results of human validation of the model’s performance. On the horizontal axis the model’s predictions are shown, while the human annotations are shown on the vertical axis. The count value represents frames in the 25-fps-video material.

As described in Section 2.6, 72 h of video material was validated to ensure model performance. The following confusion matrix (Figure 7) shows the validation results.

Table 1 shows validation results for each pig and the combined results. The combined results of all 357.388 frames in the table show an accuracy of 99.7%, precision of 99.7%, recall of 99.2% and an F1-score of 99.5%.

Event-based validation was performed using a temporal concordance analysis to quantify how well the model’s detected “drinking time” overlapped with the human-annotated “drinking time.” Results showed an exceptionally high degree of temporal overlap of 99.1%. Regarding false negative results, the fraction of time where the human observed drinking but the model did not was negligible (<0.1%). A small fraction of the concordance events (~0.9%) consisted of the model registering drinking when the human did not (false positive), typically corresponding to the “sniffing” behavior near the faucet described in the discussion. Results can be seen in Figure 2.

### 3.4. Porcine Drinking Behavior

The model was used to characterize the drinking behavior of the pigs, focusing on frequency, duration, and diurnal patterns.

**Figure 8:**
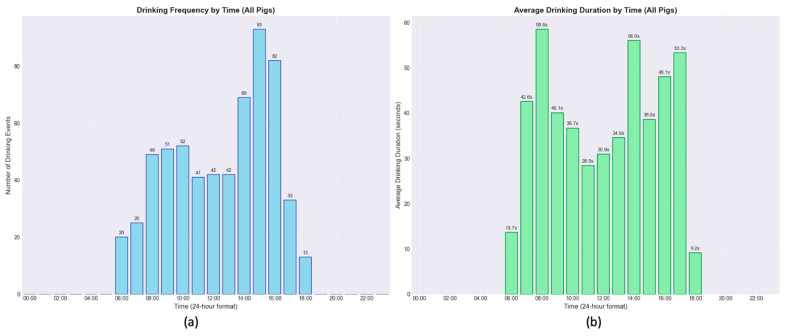
Distribution of drinking frequency (**a**) and average drinking event duration (**b**) throughout the 24 h cycle as an hourly average of the 18 days of video analyzed. The data, aggregated from all pigs, reveals that the highest frequency of drinking events occurred in the afternoon, whereas the longest average drinking duration was observed in the early morning.

The overall daily drinking patterns, aggregated across all pigs, are presented in Figure 8. Drinking frequency shows a clear bimodal distribution during the 12 h light period. A preliminary peak in activity occurs between 08:00 and 10:00, followed by a more pronounced peak in the afternoon between 14:00 and 16:00, reaching a maximum of 93 events in one hour. In contrast, the average duration of drinking events shows a different pattern. A single, distinct peak is evident early in the morning, with an average duration of 59.6 s around 07:00. For the remainder of the day, average event durations are considerably shorter, typically ranging from 28 to 56 s, even during the afternoon peak in frequency.

To investigate the correlation between feeding hours and an increase in drinking events, registration of animal caretakers within the stable was performed over 45 consecutive days using a hallway infrared sensor. Results show elevated human presence between 7 a.m. to 9 a.m. and between 12 p.m. and 13 p.m., which correlated to the announced feeding times around 7 a.m. and 13 p.m. A graph of human presence in the stable is shown in Appendix C.

Data from Figure 8 was used to calculate the power of drinking events in each hour, as well as the corresponding power percentage of the specific hour. Results are shown in Table 3.

**Figure 9:**
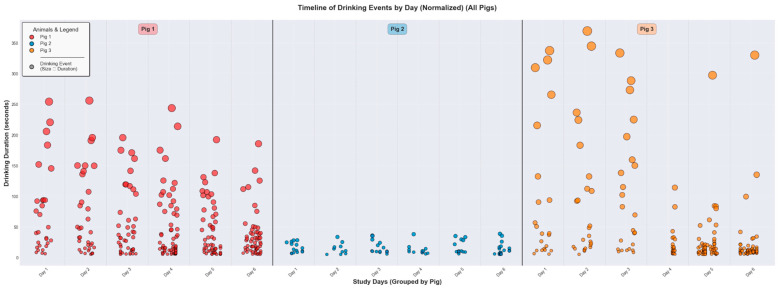
Timeline of individual drinking events for three representative pigs (Pig 1, Pig 2, and Pig 3) over their respective study periods. Each circle represents a single drinking event, with its vertical position and size corresponding to the duration in seconds. The colors distinguish between the three animals. The plot highlights significant inter-pig variability in drinking behavior. Pig 1 and Pig 3 exhibits a wide range of event durations, including many drinking events with longer duration, whereas Pig 2 displays a pattern of shorter-duration events. Only events with a duration above 5 s were considered a drinking event. The black/grey dot in the “Animals & Legend” box in the top left corner represent that the size/diameter of the coloured dots indicates the driking duration. A bigger diameter indicates a longer drinking duration. No black/grey dots exists in the figure.

Figure 9 provides a timeline of individual drinking events to highlight the unique behavioral signatures of the three pigs. Clear inter-pig variation is evident. The pattern for Pig 1 (red markers) is characterized by frequent visits to the water source, with durations ranging from brief events to prolonged sessions exceeding 250 s. Pig 3 (orange markers) shows similarly frequent drinking behavior with durations typically ranging from brief to moderate sessions up to approximately 350 s. Conversely, Pig 2 (blue markers) demonstrates a more conservative drinking pattern, with fewer visits and a much narrower, more consistent range of event durations, rarely exceeding 30 s.

These visual observations are supported by the quantitative analysis summarized in Table 4. The analysis provides daily and overall summaries of key drinking metrics, including event counts, total duration, and average duration. The data confirms the behavioral differences between the three animals. For the recorded period, Pig 1 averaged 47.7 events per day, corresponding to a total daily drinking time of approximately 38.6 min. In contrast, Pig 2 averaged 14.3 events per day for a total of 3.5 min, while Pig 3 showed an intermediate behavior, with 40.0 events per day totaling 28.3 min. This quantification confirms that Pig 1 engaged in drinking behavior most frequently and for the longest total duration, followed by Pig 3, with Pig 2 showing the least drinking activity, supporting the inter-pig variability observed visually.

To statistically verify the observed inter-pig variability, a Kruskal–Wallis test was conducted on the daily drinking event counts (*n* = 6 days per subject). A non-parametric test was chosen as the data for Pig 3 did not follow a normal distribution (Shapiro–Wilk, *p* < 0.01). The analysis confirmed a statistically significant difference in drinking frequency between the three animals (H = 11.80, *p* < 0.01). As detailed in Table 5, Pig 2 exhibited a significantly lower and more consistent activity level (Mean = 14.3, Std = 3.1) compared to the higher frequency and variability observed in Pig 1 (Mean = 47.7, Std = 12.7) and Pig 3 (Mean = 40.0, Std = 22.0). Descriptive statistics on inter-pig variation can be seen in Table 5.

## 4. Discussion

### 4.1. Main Outcome

In this study, a model was developed to recognize drinking events in videos from single-pig pens. A camera was mounted on top of each pen, and a Raspberry Pi unit was connected to receive and store the video material for on-device real-time processing. Also, the devices were able to store the data for further processing, training, and ground truth comparisons. One hundred training epochs using the box, classification, and distribution focal losses metrics were used to evaluate the model’s precision in object detection. The training metrics showed rising proficiency in the model’s object detection, validating reliable and correct object recognition with an accuracy of above 97% of all subjects of interest, which in a practical setting is sufficient. Validation of the model’s performance compared to ground truth human observations showed a high coherence, with an overall accuracy of 99.7% underlying solid performance. Deployment of the model in drinking behavior characterization of three pigs showed consistent bimodal patterns with hourly duration variation and inter-pig variation. In addition, while training was performed externally, the devices can run the models in real-time directly on the Raspberry Pi device.

While several studies have explored vision-based tracking of pig drinking behavior, our results offer significant advancements in both accuracy and accessibility. Previous recent work by Yang et al. (2018) [19] achieved 96.49% recognition using YOLOv2, and Alameer et al. (2020) [21] reported a precision of 0.98 (98%) using 3D cameras. However, these studies primarily utilized high-cost equipment or pre-recorded video for retrospective analysis. In contrast, our model achieved a higher overall accuracy of 99.7% compared to human ground truth. Most notably, we successfully deployed this high-performing model on low-cost Raspberry Pi hardware as an edge device, addressing a critical gap in the literature regarding real-time, privacy-conserving, on-device processing in research facilities. In addition, our study uniquely addresses the monitoring of minipigs in single-pig research pens—a setting largely overlooked by the existing literature. The use of edge devices increases data security, reliability, and efficiency, as video is processed locally on the gateway, thus removing the risk of video being leaked, including video of staff working on the pigs, as well as removing the need for high-speed internet connections, as well as avoiding the need for an advanced server infrastructure. In addition, current research, such as work by Zhuang et al. (2023) [22], focuses on commercial group-housed farms and often requires complex infrastructure like specialized feeding stations; our work used low-cost equipment easily available for research labs to procure and install. While Tagoe et al. (2024) [23] was the only recent study reportedly utilizing a Raspberry Pi edge device for movement tracking, they did not investigate drinking behaviors specificly.

### 4.2. Object Detection Performance

The object detection model demonstrated high performance for the classes essential to this study, particularly ‘Pig-head’ and ‘Water-faucets’ (99% accuracy each). This high level of accuracy provides a reliable foundation for the subsequent proximity-based algorithm used to infer drinking events.

The primary limitation observed was the model’s tendency to misclassify the ‘background’. The confusion matrix shows that features of the pen environment, such as bedding or shadows on the floor, were occasionally mistaken for ‘feces’ or a ‘Pig-head’. While this indicates a point for future improvement, it had a minimal impact on the primary goal of this study, as the classes of interest were distinguished from each other with very high precision.

### 4.3. Performance Accuracy

Performance metrics of the model’s performance in human observer validation include an accuracy of 99.7%, precision of 99.7%, recall of 99.2%, and an F1-score of 99.5%. These results indicate very precise model performance, which makes it very suitable for practical deployment.

False positive events occur when the pig searches/sniffs next to the drinking faucets as a behavioral habit, which causes the bounding boxes between the pig’s head and the drinking faucets to overlap. An example can be seen in Appendix A. These events do not represent a true drinking event, which is why the arbitrary limit of a minimum of 5 s was set for practical use. False negative results occur when the human observer registers a drinking event in a frame where the pig looked away for a short period. The false positive and negative results only represent 0.8% and 0.1% of frames analyzed, which has no effect on later practical implementation.

The high accuracy can be explained by the distinctive appearance of the drinking faucets, making distinguishing them very accurate for bounding box placement. Fluctuating placement in the frame of the faucets could occur between video sequences of different pens; however, this would not affect the detection of the faucets. A problem could occur if the faucet design were to change, since this would prevent the model from detecting the faucets.

### 4.4. Bounding Box and Pig Head Recognition

The system’s function relies on identifying the proximity between the bounding boxes for the pig’s head and the water faucet. This method is computationally simple, but it is an indirect measure of drinking. A key limitation is its inability to distinguish between a pig actively drinking and one that is simply resting or sniffing near the faucet. This accounts for most false positive events, where spatial proximity is correctly detected but does not correspond to the intended behavior (Appendix A). Furthermore, the top-down camera angle may lead to occasional head occlusion, potentially causing missed detections. Future work could improve specificity by using methods like pose estimation to track the snout’s position directly, offering a more reliable behavioral indicator.

### 4.5. Justification of Chosen Quantity of Video Used for Validation

The 72 h of video used for human validation was chosen to ensure a representative drinking count between different days and different pigs. In principle, validation of one day of video of one pig could be enough to validate the system. The chosen video material was randomly picked from an extensive pool of stored video material, and the chosen video was previously unseen to ensure unbiased selection.

To ensure an unbiased evaluation and prevent data leakage, the 72 h of video footage selected for validation were strictly separated from the image dataset used for model training. We cross-referenced the metadata (recording date, time, and subject ID) of the validation footage against the training dataset to confirm that no frames from the validation period were included in the model’s training, validation, or testing subsets. Consequently, the performance metrics reported in Figure 6 represent the model’s ability to generalize to previously unseen video data.

### 4.6. Drinking Patterns

To analyze the drinking pattern of experimental pigs in single-pig pens, three days of unvalidated video were additionally analyzed to ensure six consecutive days of healthy pig behavior. Three of the six days in the characterization analysis was the same video used in the validation process. Six days of video material from third pig, which was not used for validation, were analyzed to prevent validation bias in the analytical results and to allow a more extensive analysis of inter-pig variation in drinking patterns.

The drinking pattern of the pigs fluctuated between hours, as described in Section 3.4. The distribution of drinking events can be described by several factors. Firstly, the overall activity pattern described in previous studies features hours of inactivity with little to no expected drinking events [1].

Additionally, feeding times with dry feed were consistent throughout the days at around 7 am and 13 pm, which aligned well with an increased need for drinking in the following period. Taking human presence (Appendix B) and announced feeding times into consideration, naturally, pigs would have experienced increased thirst within the same hour as the pig is being fed, or the hour after the pig has been fed. 

The hours were stratified into groups depending on whether the pig has suspected thirst from feeding or whether the pigs are in non-feeding thirst-related hours. Results show an average of 10.90% in suspected feeding-related thirst hours and 5.69% in suspected. The relative power of the stratified groups in comparison to the power of all hours combined can be seen in Table 6. Here, the relative power of “thirst from feeding” hours compared to all hours combined is 141%, indicating an increase in drinking and water consumption after feeding.

### 4.7. Quantification of Water Intake

The system only aims to register drinking events as an arbitrary measurement for water intake, but it does not allow for quantification of hourly and daily water intake volume. A minimum duration of 5 s has been used to exclude drinking events that inherently do not allow for a substantial water intake.

A way to correct this problem further and to validate and quantify water intake in drinking events is to install flow sensors on the water faucet pipeline. This is neither an option in the current experimental setup nor is it a priority to add and integrate more physical hardware, since the aim of the method presented in this study is to use only camera-based data collection.

### 4.8. Inter-Pig Variation

The bedding of the pen can change during the experiments. The pen is initially bedded using hay as a bedding material, and later, sawdust is used with only a small amount of hay to satisfy the pigs’ need to sniff. Differences in bedding material could influence their need for hydration, since hay is more often used as a chewing material than sawdust.

The pigs included in the study weighed between 24 kg and 29 kg, which could have influenced their need for hydration in either direction, as a bigger pig would need more water but would be able to ingest it in bigger portions. This could explain the differences in drinking duration. The precise weight of the animals on the days of analysis is unknown, but future studies could include weight correlations for easier comparison between pigs.

Also, the three pigs lived in three different pens. The water faucets were activated by a biting mechanism. The effectiveness of the bite had an influence on the water flow, resulting in a different flow rate for each drinking visit. This could explain the inter-pig variation in drinking duration, where longer drinking events could indicate a lower water flow from the drinking faucets.

In future survival studies, each pig should act as its own control with data from baseline periods to overcome inter-pig variations.

### 4.9. Normal Variability and Illness Indication

Data used in the study was taken solely from healthy pigs; however, for later use in animal experimental setups, the need for defining an illness indicator is crucial. The variability was defined with data from the descriptive statistics presented in Table 5. Normal variability can be defined as a sustained deviation from an individual baseline median. A suggestion to define a “illness indicator” could be a to use the daily drinking median. A daily drinking median to be use as an illness indicator (ddm_illness_) could be defines as;

ddm_illness_ = baseline drinking median ± 2 × baseline median, or

ddm_illness_ = baseline drinking median ± 0.4 × daily drinking median

The daily drinking median for illness could be equivalent to more or less than two times the standard deviation of the baseline drinking median or equivalent to an increase or decrease of 40% of the daily baseline median.

### 4.10. Image Pre-Processing in a Fixed Setup

We decided to exclude image pre-processing from our analysis based on the specific conditions of the study and the capabilities of the chosen algorithm. The highly controlled environment, with its static camera and regulated lighting, provided consistently high-quality visual data, mitigating the need for common correction techniques [31]. This stable data stream is well-suited for the YOLOv11n architecture, which is inherently robust to the minor image variations present in this setup [32]. Furthermore, by operating directly on raw frames, we avoid introducing potential processing artifacts that could negatively affect the model’s performance.

However, this methodological choice significantly limits the model’s generalizability. The system is tailored specifically to this research facility’s conditions. Its performance would likely be much lower in environments with variable lighting, different pen configurations, or the presence of multiple animals. Therefore, the current model should be considered a specialized and feasible tool for this setup, where finer corrections would allow for general scalability to other research experiments using single-housed minipigs. Permanent installations would require minimal maintenance, and technical or conceptual improvements and adjustments could be performed remotely. The setup is not a suitable solution for diverse agricultural settings without substantial further development and training on more varied data.

### 4.11. Future Development

#### 4.11.1. Water Bowls

Taking animal welfare into account, detecting sickness early is essential. Experimental animals can become unwell to different degrees because of interventions made in research settings. During experiments, pigs could be offered water bowls to improve water availability, as seen in Appendix C. This results in non-detected drinking events that are not registered for later drinking pattern analysis.

Future work should, for this reason, include object detection of water bowls. This improvement would allow the system to analyze how drinking behavior is influenced by sickness and changes in health over time. Animal caretakers and researchers thereby gain more knowledge of the pig’s current health state to treat potential complications and intervene faster.

#### 4.11.2. 24-Hour Monitoring

Pigs are known to engage in nocturnal activity, but this cannot be analyzed with classical video monitoring, since the room is totally dark. To include nighttime behavior in the overall characterization of the pigs, different add-on detection methods could be added in future developments of the system. Firstly, infrared cameras placed over the pen could give valuable knowledge on nighttime activity levels and drinking events. The data could easily be validated using daytime measurements for comparison. Secondarily, microphones would add information on pig sounds and drinking events, since activation of the drinking faucet makes a distinctive sound. Characterization of pigs’ welfare using sound has been performed in a previous study [33], but in a practical setting, identification of specific pigs when recording sound in a multi-pig pen room would be the main obstacle.

#### 4.11.3. Early Warning System

Real-time deployment of monitoring systems is essential in work towards using machine learning-based systems to track changes in research animals’ behavior and welfare in real time. Pigs are complex animals, and classification of their current health state requires detailed multi-nodal information from monitoring systems, if such systems should contribute to or replace human observation methods. In the future, information on their drinking behavior combined with analysis of their activity, nocturnal behavior, and fecal excretion and urine output could create a complex observation system. Such a system would allow for detailed analyses of changes in the pigs’ health status, which would warn personnel of suspected declines in health. Eventually, a system like this could be used as a real-time alternative to human observation methods.

## 5. Conclusions

A novel machine-learning-based software model for identifying drinking duration and event count was developed, validated, and tested using 72 h of video of healthy Göttingen minipigs used for research. The software relied on a YOLOv11n object detection model to detect “water-faucet” and “pig head” and the proximity of the two to register a drinking event. The model was also used to characterize the drinking behavior of three pigs using 216 h of video material.

Object detection results demonstrated a very high accuracy of all primary objects of <97%. Validation of the system showed an overall accuracy of 99.7%, with a precision of 99.7%, recall of 99.2%, and F1-score of 99.5%. Behavioral analysis of the drinking pattern of three pigs over three days revealed a bimodal pattern consistent with their activity pattern. Clear inter-pig variation in event count, duration, and average drinking time was observed.

The system represents a highly accurate method of automatic, light-dependent detection of drinking behavior in experimental pigs in single-pig pens, which is not suitable for multiple-pig pen setups. Further adjustments using infrared cameras would allow for 24 h observation. Infrastructure from the developed model could be included in a more complex system for surveillance of experimental animals with an aim to improve animal welfare in animal experiments.

## Figures and Tables

**Table 1:** Performance results with the performance parameters (column labels) of the two pigs and combined results (row labels).

Pig	Accuracy	Precision	Recall	F1-Score	Total Frames
Pig A	0.999	0.996	0.998	0.997	185,782
Pig B	0.996	0.998	0.996	0.992	171,606
Combined	0.997	0.997	0.992	0.995	357,388

**Table 2:** Metric descriptions.

Metric	Description
Train/box loss	This graph shows the loss associated with the bounding box predictions during the training phase. The *y*-axis measures the loss magnitude, which ideally decreases over epochs (*x*-axis), indicating the model’s improving accuracy in locating objects.
Train/cls loss	Displays the classification loss during training, measuring how well the model identifies the correct class labels of the objects. The declining curve suggests improving classification accuracy.
Train/dfl loss	Shows the distribution focal loss during the training phase, which focuses on enhancing the model’s accuracy in localizing the exact position of objects. Lower values indicate better localization capabilities.
Metrics/precision (B)	This metric indicates the precision of the model during training, calculated as the ratio of true positive predictions to the total positive predictions made by the model.
Metrics/recall (B)	Reflects the recall rate during training, which measures the model’s ability to identify all actual positives. Higher recall values suggest that the model misses fewer actual positive cases.
Val/box loss	Like the train box loss but for the validation phase. It assesses the model’s ability to generalize bounding box predictions on unseen data.
Val/cls loss	Represents the classification loss during validation. A decreasing trend here is crucial as it signifies effective generalization in classifying new, unseen data.
Val/dfl loss	Mirrors the train distribution focal loss but evaluated on the validation dataset. It checks the model’s performance in object localization under validation conditions.
Metrics/mAP50 (B)	Depicts the mean average precision at the 50% Intersection over Union (IoU) threshold during training. This metric assesses the accuracy of the object detector by considering both precision and recall at a moderate IoU threshold.
Metrics/mAP50-95 (B)	Shows the mean average precision across a range of IoU thresholds from 50% to 95% during training. This comprehensive metric evaluates the object detector’s performance across varying levels of detection difficulty, reflecting its effectiveness in various scenarios.

**Table 3:** Summary of “drinking power” calculations for each hour when summing up all analyzed days.

Hour	Events	Avg. Duration (s)	Power (s)	% of Total Power
6	20	13.7	274.0	1.08
7	25	42.6	1065.0	4.20
8	49	58.6	2817.4	11.32
9	51	40.1	2045.1	8.06
10	52	36.7	1908.4	7.53
11	41	28.5	1168.5	4.61
12	42	30.9	1297.8	5.12
13	42	34.6	1453.2	5.73
14	69	56.0	3864.0	15.24
15	93	38.6	3589.8	14.16
16	82	48.1	3944.2	15.55
17	33	53.3	1758.9	6.94
18	13	9.2	119.6	0.47
Overall	612	41.44	25,359.9	100

**Table 4:** Tabulated summary of daily drinking statistics for all three pigs. Key metrics include daily event counts, total drinking time, and average event duration. Std defines standard deviation from the average drinking duration, while “First” and “Last” refer to the clock hour of the first and last drinking event on the particular day. The data quantifies the significant differences in drinking volume and frequency between the three animals.

Day	Events	Total (s)	Total (min)	Avg (s)	Max (s)	Std (s)	First	Last
**Pig 1**								
1	29	2133.4	35.6	73.6	254.2	70.7	7.5	16.2
2	35	2272.2	37.9	64.6	255.9	66.5	7.3	17.0
3	44	2285.2	38.1	51.9	195.5	53.9	7.1	17.1
4	64	2828.5	47.1	44.2	243.7	53.2	6.8	17.2
5	55	2327.3	38.8	42.3	192.2	43.4	7.2	17.9
6	59	2041.9	34.0	34.6	185.6	36.2	7.0	17.1
Average per day: 47.7 events|2314.7 s|38.6 min								
**Pig 2**								
1	15	240.5	4.0	16.0	28.3	8.1	8.1	17.2
2	10	145.5	2.4	14.5	33.6	9.3	7.3	18.2
3	15	244.6	4.1	16.3	35.8	10.1	9.3	18.0
4	12	151.7	2.5	12.6	38.1	8.7	6.8	17.6
5	14	231.7	3.9	16.6	35.0	10.5	9.1	15.2
6	20	261.6	4.4	13.1	38.7	9.9	8.1	16.7
Average per day: 14.3 events|212.6 s|3.5 min								
**Pig 3**								
1	22	2108.3	35.1	95.8	337.4	114.4	8.1	8.1
2	25	2235.6	37.3	89.4	369.4	105.0	9.3	7.3
3	24	2388.9	39.8	99.5	333.5	100.1	10.1	9.3
4	27	560.5	9.3	20.8	114.1	24.8	8.7	6.8
5	73	1563.7	26.1	21.4	297.3	37.1	10.5	9.1
6	69	1345.6	22.4	19.5	330.1	42.5	9.9	8.1
Average per day: 40.0 events|1700.4 s|28.3 min								

**Table 5:** Descriptive statistics and test results from a Shapiro–Wilk test for normality and a non-parametric Kruskal–Wallis for inter-pig comparisons. The tests show Pigs 1 and 2 followed a normal distribution, while Pig 3 did not. Inter-pig comparison confirmed statistically significant differences between the pigs that were not solely due to daily variation.

Subject	Median (Events/Day)	Median	Std. Deviation	Range (Min–Max)
Pig 1	47.7	49.5	12.7	29–64
Pig 2	14.3	14.5	3.1	10–20
Pig 3	40.0	26.0	22.0	22–73
Overall	34.0	28.0	19.8	10–73
**Statistical Test**	**Test statistics**	***p*** **-value**	**Result**	**Note**
One-Way ANOVA	F = 6.97	0.007	Significant	(Assumes normality)
Kruskal–Wallis	H = 11.80	0.003	Significant	(Preferred method)

**Table 6:** Characterization of drinking power when stratified hours into suspected thirst from feeding or non-feeding related thirst.

Hour Characterization	Hours	Avg. Power (% of the Day)	Relative Power
Thirst from feeding	8 + 9, 13–15	10.90	141%
Non-feeding related thirst	6, 7, 10, 11, 12, 16, 17, 18	5.69	74%
All	6–18	7.69	100%

**Figure A1:**
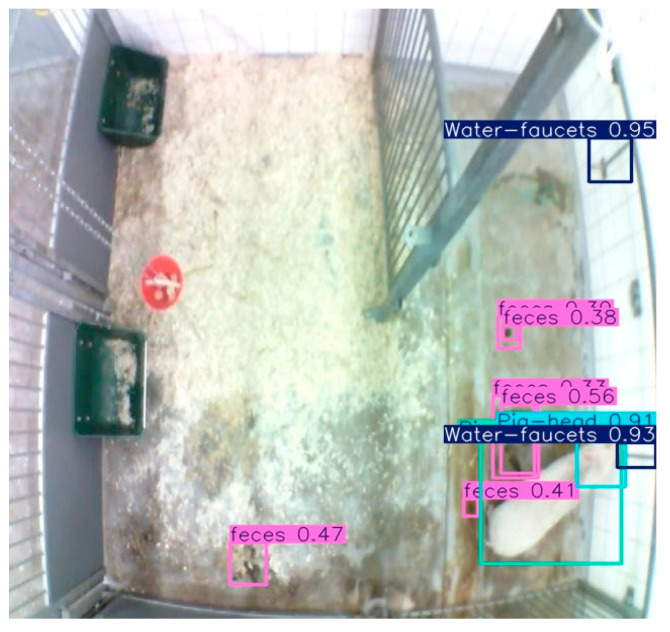
A visual example of a false positive registration of a drinking event. The light blue/green box represent the bounding box of the pig, the pure light blue box represents the bounding box of pig head, the dark blue boxes represent the bounding boxes of the water faucets, and the pink boxes represent the bound box of feces on the floor (not relevant for this study). The pig is seen looking past the water faucet without drinking from the water faucet, but the model (correctly) detecting an overlap of the bounding boxes between the pig’s head and water faucets.

**Figure A2:**
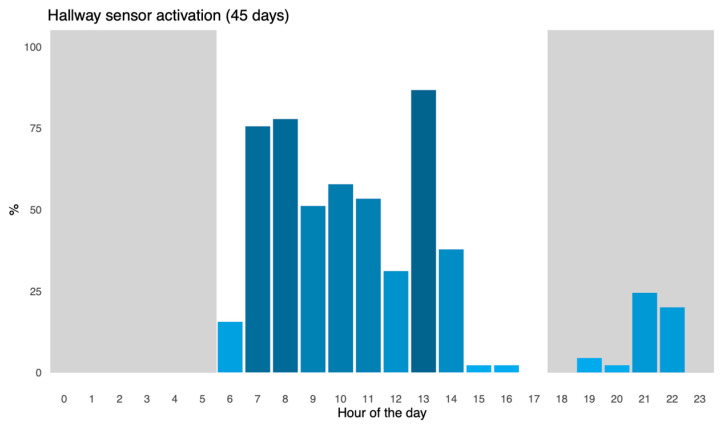
Each column represents how many of the 45 days, activity on the infrared hallway sensor (Motion and Light Sensor P2, Aqara, Shenzhen, China) in the specific hour was registered (in percentage), as an indirect measure for human presence in the stables as part of the daily management of the experimental animals. Lighter blue indicates a lower percentage, while a darker blue indicates a higher percentage. A total of 45 days of data was used in total. Data showed a marked increase in human presence from 7 a.m. to 9 a.m. and from 12 p.m. to 13 p.m., which corresponded to the mentioned feeding times.

**Figure A3:**
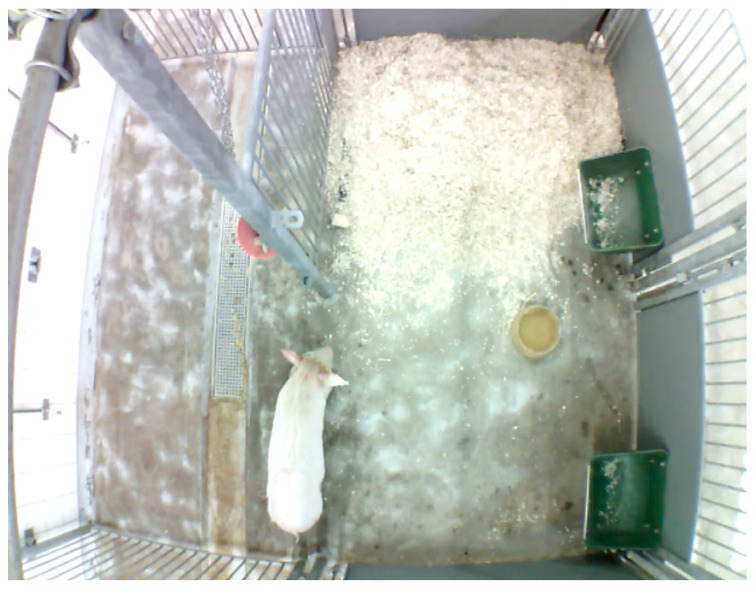
An example of a water bowl placed in the pen (yellow structure on the right side of the pen). Drinking from water bowls is not registered, so further improvements would include object recognition of water bowls in the frame.

## Data Availability

The data presented in this study are available on request from the corresponding authors.
